# Guidance for Use of Artificial Intelligence in Community Pharmacy Practice: Perspectives and Needs of Pharmacists in Ontario, Canada

**DOI:** 10.3390/pharmacy14020057

**Published:** 2026-04-01

**Authors:** Zubin Austin, Paul Gregory

**Affiliations:** Leslie Dan Faculty of Pharmacy, University of Toronto, Toronto, ON M5S 3M2, Canada; paul.gregory@utoronto.ca

**Keywords:** artificial intelligence, community pharmacy, pharmacy practice

## Abstract

Background: As Artificial Intelligence (AI) proliferates in society, community pharmacists must make decisions as to how to responsibly adopt this technology in their practice. Currently, there are few regulatory requirements or tools to support pharmacists in ensuring safe and ethical integration of AI in their work. Methods: An exploratory qualitative study of community pharmacists in Ontario, Canada was undertaken to examine their needs for guidance, regulation, and support in adopting AI in their practice. Results: Semi-structured interviews with 24 community pharmacists were undertaken to the point of thematic saturation. Constant-comparative analysis highlighted three key themes: (a) currently, AI is being used in unstandardized and unregulated ways; (b) pharmacists desire guidance or regulation focused on patient safety considerations; and (c) in the absence of regulation, ad hoc informal decision making is occurring. Conclusions: With or without formal regulation, AI is being adopted in pharmacy practice. Current reliance on informal network support without clear regulatory guidance raises concerns for pharmacists regarding patient safety and their work as professionals.

## 1. Introduction

Artificial Intelligence (AI) is generally described as technology that is capable of performing activities most typically associated with human cognition or potential [1,2,3]. AI systems are generally categorized in one of three ways [4,5]. Human-in-the-loop (HiL) AI requires human oversight and approval, with AI simply providing recommendations or data, with humans having ultimate decision-making control. Within pharmacy, HiL AI systems are often used to alert clinicians to potential drug–drug interactions, or allergy notifications [6]. In contrast, human-out-of-the-loop (HoL) AI cedes decision making functions to the AI with no requirement for humans to review or approve actions prior to their implementation. Within pharmacy, HoL AI systems are being deployed to monitor inventory and automatically reorder when thresholds are met [6]. A third variant, human-on-the-loop (HonL) AI, represents an intermediate state in which human beings are still required to take responsibility for final decisions and actions recommended by AI, but these humans become sufficiently de-skilled or overly reliant on AI so they do not in reality exercise independent critical judgement. Instead, in HonL AI, humans automatically agree to AI recommendations because it is a system requirement, but are not truly engaged in reflection, critical questioning, or exercise of professional judgement. Any HiL AI system can operationally function as HonL AI based on how the pharmacist actually responds [6].

Today, AI is ubiquitous and used by countless billions of people every day—whether they even know they are using it or not [7]. For example, most smartphone technologies today embed AI in every process from the calendar function to search engines. Popular media services such as Netflix (Netflix, Los Gatos, CA, USA) rely on AI to generate recommendations for consumers to continue to engage them in that service. While fully autonomous self-driving cars are still not widely deployed, many safety features on both electric and conventional internal-combustion engine vehicles rely on AI to support safe driving. Importantly, everyday use of AI by average people is rarely—if ever—disclosed explicitly, and there are few circumstances where explicit informed consent for AI use in consumer technologies is sought or required [8]. This is despite the question of what happens to the data that consumers are ceding using the technology itself. In using AI-powered technologies, consumers are actively contributing to the datasets that train the AI itself and make it more powerful than ever. Questions regarding privacy, confidentiality, safe storage and responsible use of this data are generally not discussed [8,9]. Instead, somewhat naively and entirely willingly, most consumers simply use consumer electronic devices in ways that may expose them to data mining or other practices that feed the training of AI with limited or no regulatory oversight [9,10].

The everyday experience of AI by consumers is mirrored by the expansion of AI in the practice of healthcare professionals. Whether patients know it or not, AI has penetrated healthcare work in diverse ways [11]. From ambient listening systems that use AI to transcribe patient–practitioner conversations to diagnostic and prescribing decision support tools, AI has become part of most practitioners’ day-to-day work—sometimes in ways that they are not fully able to articulate [11,12]. In some cases, organizations formally and intentionally deploy AI systems using managerial systems focused on safe and effective implementation, but in other cases well-intentioned practitioners may simply use their personal cell phone to use commercially available AI products like ChatGPT version 5.1 (Open AI, San Francisco, CA, USA) or GoogleLens (Google, Mountain View, CA, USA) or translation programs, to expedite their work, not fully realizing the privacy, security, quality, and safety implications of what they are doing [12,13]. Questions regarding transmission, disposition, storage, and use of data voluntarily given to AI systems this way are significant [13,14,15].

Anecdotally, it appears that many health professionals—including pharmacists—have mixed feelings regarding the proliferation of AI in both their professional and personal lives [6]. On the one hand, there is the promise and potential of machine learning opening astonishing future opportunities for humanity, and, as scientifically trained professionals, pharmacists understand the power of progress to improve human life. Within pharmacy, the potential positive benefits include enhanced medication safety, drug–drug interaction screening, workflow optimization, and better inventory management [6]. On the other hand, both as citizens and professionals, pharmacists recognize the tendency of the big technology companies to underwrite the development of AI to overhype their products and to generate innovation without sufficient attention to important public protection safeguards and guardrails. The experience of social media, including ubiquitous access to platforms such as Facebook (Meta, Menlo Park, CA, USA) or X (X Corp, Bastrop, TX, USA) has left many individuals feeling cautious or cynical about new technologies, particularly as socio-political impacts of these platforms are increasingly viewed as harmful and negative [16]. These conflicting positive and negative narratives surrounding the emergence of AI leave many individuals simply feeling confused and overwhelmed, and powerless to understand it and use it in a responsible and ethical manner.

Subjective feelings aside, the reality today is that pharmacists are being thrust into the forefront of the use of AI in clinical practice, whether they choose to engage or not. Patients already come to pharmacies with AI-generated diagnoses and treatment plans that must be managed in clinical interactions. Pharmacy staff and students use sanctioned and unsanctioned AI to manage workload and enhance efficiency in day-to-day practice, despite concerns it may have with respect to safety, efficacy, and their own deskilling. Health systems and employers are adopting AI as a way to manage staff shortages, reduce costs, increase efficiency, and manage administrative and managerial issues.

This leaves frontline pharmacists in a challenging situation with respect to how they—as clinicians and patient-care providers—should be engaging with AI. Few pharmacists have a technical or engineering background to truly understand the mechanics of AI and how it works. While some interested pharmacists may learn useful skills such as prompt-engineering that can enhance the quality of AI outputs, the majority are likely using AI in a passive, non-critical, and unquestioning manner, despite having personal concerns regarding this approach. Most pharmacists will be aware of AI’s documented deficiencies with respect to hallucinations [17] and sycophancy [18]. Hallucinations are generally described as situations in which an AI model will produce an output that appears reasonable or plausible, is presented with great confidence and certainty, but contains false or meaningless information. In some cases, AI appears to actually invent facts or information sources. Its thought that hallucinations are not simply a “bug”, but actually a feature of AI itself; the large language models that undergird AI are based on statistical relationships between words in an attempt to allow AI to generate coherent text and images [17]. Where gaps in the training data exist, AI is actually trained/programmed to use forms of inductive cognition—literally, to “make things up”, so as to confirm the general trend of the pattern in a way that reinforces that pattern. In this way, hallucinations are a form of machine-learning confirmation bias [17]. A second issue that has been described is the tendency towards sycophancy, especially where AI-driven chatbots are used in customer service, counselling, education, or psychotherapeutic context. Sycophancy describes the process by which an AI chatbot may excessively and unrealistically agree, flatter, or mirror a user’s beliefs of opinions [18]. Similar to the issue of hallucinations, sycophancy may be a feature of AI, rather than a simple “bug” that can be fixed. AI generates sycophantic responses precisely to continue to engage with the user, and, in the process, sacrifices what could be reasonably construed as objectivity, facts, and truths for the purpose of gaining approval by the end user [18]. In the context of therapeutic chatbots, this has produced dangerous outcomes in some cases—recently publicized stories of chatbots agreeing that young people should commit suicide or commit homicide represent extreme cases of sycophancy, but even in less impactful situations (such as smoking-cessation chatbots) there have been reports of disturbing and incorrect recommendations being generated (such as encouraging simultaneous use of nicotine patches and smoking cigarettes).

Hallucinations and sycophancy are but two examples of the concerns citizens and health professionals have regarding use and reliance on AI in clinical practice. Both represent the broader concern associated with the structure of large language models and training of AI agents themselves [1,2,3,4]. The acronym “GIGO” (Garbage In, Garbage Out) describes the problem of flawed/incorrect data, or outright misinformation or disinformation, contaminating the datasets that are used to train AI and give it its power. In some fields—for example, nutrition sciences—there are significant concerns that much, and perhaps the majority, of information on the Internet that is and could be used to train AI models is flawed, wrong, or deliberate misinformation. If this is the dataset upon which AI models are built, its outputs, recommendations, and chatbot-driven conversations will be deeply problematic—yet they appear confident, well-informed, and true to naïve end users. Pollution of the information ecosystem upon which AI is built is emerging as a significant socio-political threat, as it starts to threaten the foundations of what we, as a civilization, know and believe to be objective truths, and this process may be accelerating significantly in this era of AI.

For these reasons, many of those who were involved in the initial development of AI have raised alarms regarding its growth and proliferation, particularly in the context of enormous for-profit technology companies [19]. While the promise and potential of AI seem enormous to the point of magical, the risks and threats appear ominous and potentially civilization-altering. While such massive stakes and consequences may be fodder for dystopian science fiction, the reality today is that pharmacists must navigate all these conflicting and confusing issues with minimal guidance and support in determining whether and how they can or should be integrating AI into their daily practice in a responsible and ethical manner.

The objective of this research was to examine the perspectives of community pharmacists in Ontario, Canada regarding responsible and ethical adoption of AI in their practices. This study sought to better understand their questions, concerns, and decision-making processes as a way of characterizing fundamental issues of understanding of the technology and its role in practice. This is an important step in identifying needs and opportunities for educators, regulators, employers, unions, and others, to provide education and support to enhance safe and effective adoption of this rapidly evolving technology in practice.

## 2. Materials and Methods

Given its relatively recent commercialization and rapid evolution, there is currently limited research examining community pharmacists’ use of and attitudes towards AI in their day-to-day practice. As a result, a qualitative exploratory-research method was selected as being most appropriate to study this issue [20]. Semi-structured interviews with community pharmacists were identified as the most appropriate research method to encourage honest disclosure and in-depth conversation [21]. Focus groups were also considered for this study but ultimately not selected, as they may not be as conducive to full and honest conversation as semi-structured one-on-one interviews [21].

To ensure quality and rigour of the research, the consolidated criteria for reporting a quality research (COREQ) checklist [22] was used to guide all aspects of the research process, from initial framing of research objectives through to methods, data collection and storage, analysis, write-up and researcher reflexivity. For data analysis, a constant comparative method was selected in which all transcript data from interviews was read by each researcher and then coded and categorized independently [23]. Researchers then conferred and compared and contrasted their coding and categorization to identify common themes, based on their independent reading of transcripts [23]. These themes were then confirmed, modified, or rejected, based on reading and review of subsequent interview transcripts, and also analyzed independently and then compared. Data saturation was defined as the point of research conclusion [24]. Saturation occurred at the point where both researchers independently confirmed that no new codes, categories or themes emerged from analysis of a new transcript; to confirm, two additional interviews, transcript examination, and analyses were undertaken following the point at which saturation was declared, to ensure quality and integrity of data collection and analysis [24].

The researchers involved in this study have extensive experience in health services and qualitative exploratory research of this sort, but have no particular interest or expertise in AI. They have extensive experience in pharmacy practice research, and, in particular, working with community pharmacists. Neither researcher is a data scientist or computer scientist; one of the researchers (ZA) is a licensed pharmacist with over 30 years’ experience in the profession, while the other (PG) is an information professional with expertise in qualitative research.

The participant pool of interest in this research was community pharmacists involved in the provision of direct patient care (as opposed to managers, owners, or others who supported the business of pharmacy rather than the delivery of care to patients). Inclusion criteria for this study were: (i) a minimum of 2 years’ experience working in community pharmacy (in order to ensure sufficient exposure to practice and patients, to mitigate the learning curves of novices); (ii) a minimum of 24 h weekly in direct patient-care activities (to ensure sufficient exposure to patients and community pharmacy practice itself); and (iii) English-speaking (due to linguistic limitations of the research team). Hospital and other types of pharmacists were excluded from this study, in order to maintain a focus on the community variant of practice. Regulated pharmacy technicians were also excluded from this study, as their scope of practice and activities are distinct from pharmacists. The geographic focus for this study was Ontario, Canada. Ontario is the largest province in Canada, home to approximately 15 million people. As of mid 2025, there were approximately 19,000 registered pharmacists in Ontario; roughly 60% of these individuals declared community pharmacy as their primary site of patient-care practice [25].

Within Ontario, there are many different types of pharmacies, ranging from small, independent businesses where the owner/operator is also the manager and the only pharmacist who delivers patient care, to corporate pharmacies employing multiple pharmacists, to “big box” pharmacies in enormous grocery store chains. Each type of pharmacy may have a different philosophy and approach to patient care. Further, as a large and geographically diverse province, Ontario encompasses world-class cities with populations of over 8 million people, suburban regions, smaller towns, and rural/remote communities where the pharmacy may be the only health care centre available. Capturing this variation in geography and practice type is challenging in exploratory research such as this; sampling to ensure true representativeness of all types and locations of pharmacy would result in an infeasibly and unrealistically large number of interview participants that would be required to participate. Since interviewing to thematic saturation was identified as the most appropriate method for this research, stratification of the interview pool was not undertaken; instead, saturation was achieved and demographic characteristics of the respondent pool are presented (see Table 1) to allow readers to better understand who participated and who did not.

Recruitment for this study was undertaken in several stages. An email was sent to all registered pharmacists who had previously consented to be contacted to be involved in pharmacy-practice research projects. This email contained preliminary information about the objectives of the study and the methods that would be used, including inclusion criteria and time requirements. Interested pharmacists were invited to respond to the email if they had further questions or wished to learn more. Pharmacists interested in learning more were provided with informed consent documentation that outlined details of the study and encouraged them to ask further questions to confirm their understanding and interest in participating. Informed consent for participation was obtained from all subjects involved in the study, based on a research protocol approved by the University of Toronto Research Ethics Board.

A semi-structured interview guide was developed, pilot tested, refined, and finalized for use in guiding data collection. The guide initially consisted of 14 questions/prompts (some with sub-prompts), and took 65 min to complete during pilot testing. Feedback from 3 pilot participants facilitated modification of the guide and reduction of question density; the final version of the protocol used for this study and approved by the research ethics board consisted of 9 questions (some with sub-prompts) and took approximately 40 min to complete (see Table 2).

All semi-structured interviews were conducted virtually, using the Zoom 6.7.8 platform (Zoom Communications Inc., San Jose, CA, USA). This platform allows for simultaneous generation of transcripts of recorded conversations using an AI agent to enhance accuracy. This was used to facilitate preliminary generation of transcripts; all transcripts were reviewed by the interviewer and modified as required, to more closely conform to the interviewer’s experience of the interview. Prior to data analysis, each participant was offered an opportunity to review their own transcript and make comments and amendments or withhold data, based on their review.

Once transcripts were reviewed and cleaned by the interviewer and reviewed and accepted by the participant, data analysis using the constant comparative method was undertaken. Both researchers involved in analysis utilized NVivo v15.3 (Lumivero, Burlington, MA, USA) for data storage, management and analysis. This version includes an AI agent that can be engaged to summarize documents (generating a 1–2-page precis of the entire transcript, in order to provide coders with a more efficient way of differentiating between different transcripts), suggest preliminary categories (based on semantic analysis of the transcript text), and provide AI-generated summaries for analytical frameworks and coding structures. This functionality was enabled and used for this research in order to increase efficiency of the process. It was only used by researchers during the initial data analysis phase; all final individual decisions regarding categories, codes, and themes were made by each researcher independently, and then collaboratively, as prescribed by the constant-comparative analysis method. For this study, AI was used to support the research and enhance operational efficiency using human-in-the-loop methods, where final decision-making authority rested with the human research-team members. Throughout this process, specific transcript data were referenced constantly, in order to ensure a clear evidentiary basis for identified codes, categories, and themes. In the constant comparative research method, where discrepancies or disagreements in coding, categorizing or theming arose between research team members, discussion and consensus were required in order to advance the analysis, as outlined in the COREQ checklist.

This study received ethics board approval from the University of Toronto, Canada (protocol 46033 approved 2 January 2024). It was deemed “low risk”, research given the minimal threat of vulnerability faced by educated professional participants who were not discussing sensitive patient-specific information. This manuscript reporting findings of the research was entirely generated by human researchers using commercially available word processing software, with no assistance from an AI agent.

## 3. Results

A total of 52 individuals responded to the initial email inviting expressions of interest to learn more about this study. Based on previous experience with pharmacy-practice research projects such as this, this was deemed to be enough potential participants, and no second or subsequent emails were sent. Of the 52 who initially responded to the email and were sent documents providing further details regarding the study (including informed-consent provisions), 31 indicated interest in participating. When contacted by the research team to answer further questions and complete informed consent, 28 individuals were ultimately enrolled in the study. Of these 28, 4 individuals dropped out, due to a variety of personal and professional circumstances, and did not complete the interviews. In total, 24 participants completed interviews and their transcript data was reviewed and analyzed for this research. All participants were offered the opportunity to review their transcripts; 8 individuals accepted this invitation, and no changes, amendments, modifications or removal of data requests were received. Demographic characteristics of participants are presented in Table 1.

Thematic saturation was assessed independently by both coders in this study as the point at which no new additional information was gleaned from interviews. Both coders independently determined saturation after review of the 21st and 22nd interview transcripts. The iterative nature of constant comparative coding used in this study was coupled with frequent check-ins (after every five transcripts had been independently analyzed) between the coders, to confirm understanding of themes and definition of codes. After the first five transcripts had been analyzed, discordant coding and categorizing was noted, and a discussion between coders was undertaken to develop a coding-structure dictionary to confirm common understanding and labelling of codes. After the next five transcripts were analyzed, more than 50% of codes were aligned between reviewers, and additional clarifying discussions were undertaken. After the 15th transcript was analyzed, inter-coder consistency was greater than 85%, demonstrating strong agreement and alignment between coders. By the 20th transcript, inter-coder consistency of greater than 95% was achieved, and two additional interviews were undertaken to confirm thematic saturation had been achieved. The 21st and 22nd interviews were analyzed, also demonstrating 95% with codes and identified themes, at which point both reviewers were comfortable in declaring thematic saturation. As per the study protocol, two additional interviews were completed and transcripts analyzed, for a total of 24 in total.

Constant-comparative analysis highlighted three key themes: (a) currently, AI is being used in unstandardized and unregulated ways; (b) pharmacists desire guidance or regulation focused primarily on patient safety considerations; and (c) in the absence of regulation, ad hoc informal decision making is occurring. These three themes were distilled from an initial bank of 62 different codes determined by both independent coders. Through the process of iterative coding and cross-checking between coders, these 62 codes were reduced to 18 codes following analysis of the first five transcripts, then further reduced to six themes following analysis of the first 10 transcripts. Following check-in after the 15th transcript, a super-code was identified focused on the participants’ need for strong regulatory leadership in defining trustworthy AI in pharmacy. The final three themes described below were assembled under this super-code, and were agreed upon by both reviewers, following the 21st and 22nd interviews.

*Theme 1: AI is already being used in unstandardized and unregulated ways*

*“Well, you just sort of teach yourself how to use it I guess? You fiddle with it, try it out in situations where you kinda already know the answer, then go from there. It doesn’t seem like a good system to me at all, because everyone, well they’re just learning by trial and error. Sometimes if you have kids or students in the pharmacy they can show you a few tricks, but as it stands now it makes me really nervous that we are all using this power technology but don’t know really what we’re doing with it.”*

*“It seems strange to me that [AI] is being unleased on the world and everyone treats it like a toy. It’s a game. No one needs to take a course or pass an exam or do anything to show they really know how to use this technology. And that’s a problem in my opinion—if we aren’t using it properly, how do we know it’s safe or effective? Everyone I know uses AI but we all do it so differently and based on—I don’t know—so it makes me worry are we all getting different answers?”*

*“I’ve actually tried this and its scary to me. I can enter the exact same question into [ChatGPT] as you but I get a completely different answer using my phone or computer than you do. And it’s not only whether it’s the free version or the paid version. Isn’t that something we should all be worried about? Shouldn’t this be standardized, I mean if it is the same question shouldn’t we be getting the same answer? But we don’t, every time I’ve tried this with someone else we get different answers. I think [the regulatory body] should be way more concerned about this.”*

At the time of this study, participants noted there were highly personalized, unstandardized ways in which AI was being used by themselves and others in their day-to-day practice, and that this was a source of concern. All participants in this study were familiar with commercially available general AI products (such as ChatGPT or Gemini (Google, Mountain View, CA, USA)), as well as pharmacy-specific AI-powered products, but expressed considerable discomfort in their knowledge and ability to actually assess quality, safety, efficacy and efficiency characteristics of individual products. All participants in this study indicated that, at some time, they had used personal devices (mainly mobile phones) within the pharmacy environment to access AI (typically ChatGPT) to rapidly find answers to questions; unless prompted by the interviewer, no participants expressed concerns regarding privacy, confidentiality of patient information, or other concerns associated with this activity. In all cases, participants noted that they had “played” with AI on their personal devices as a way of gaining familiarity with its capabilities, and that in the context of a busy clinical practice where access to fixed hardware terminals in the pharmacy was constrained due to workload, it was simply easier, faster, and more efficient to use a personal device to rapidly access information. When prompted to discuss this behaviour further, most participants noted that there were no formal prohibitions on doing so, and that in a pre-AI era they would sometimes or regularly use personal phones to rapidly find information to support their patient-care activity.

When prompted by the interviewer, most participants expressed limited understanding regarding differences between “free” and “paid” (including subscription) versions of commercially available AI products. Only two of the participants in this study had subscription versions (both of ChatGPT); these individuals commented on the surprising differences they encountered in using this paid version, and highlighted in particular the enhanced quality and depth of responses they felt were generated using the subscription service.

Most participants expressed confusion regarding what level of AI may or may not be present in the dispensing and clinical-support software used on hardware and computer terminals provided by their employers. Most believed that AI was being used for, or supported, functions related to drug information, documentation, chart audit, clinical decision-making support, allergy verification and drug–drug interaction detection, but were unsure, and could not describe this any further. Participants also expressed uncertainty regarding the presence or absence of AI elements in other commonly used applications. For example, the GoogleLens app allows mobile phone users to take a picture of a product and then identify its manufacturer and source. This app—on a personal mobile phone—was used by many participants in this research to facilitate identification of unknown pharmaceuticals (for example, a person from another country asking for a similar medication to something they bought abroad). Most participants expressed a lack of awareness or understanding regarding the role of AI in powering apps such as GoogleLens or what implications may exist from using this on a personal device in a professional problem-solving context.

All participants in this study noted the current lack of regulation, standardization, and education focused on AI in pharmacy practice. All participants described themselves as self-taught with respect to AI, and all of them indicated this raised personal concerns for them regarding the quality, accuracy, and effectiveness of their skills related to AI use in pharmacy. All participants also noted their concerns for patient safety and quality of care, and strongly endorsed a greater role for educators and regulators in helping professionals navigate this technology more appropriately and responsibly.

*Theme 2: Pharmacists desire guidance or regulation focused primarily on patient safety considerations*

*“If we are going to be using AI—if we are expected to use it—well then someone needs to help us to use it safely, effectively. I know I have so many questions—what about privacy of patient data, what about hallucinations or if it gives the wrong answer, what if I don’t agree with the answer it gives? There’s so many situations that we have to be prepared for and it is so frustrating that no one—not the [licensing body] or employers or anyone—gives us any kind of guidance.”*

*“Well it’s mainly about safety, right? What’s safe for the patients? Everyone knows about the hallucinations, you know that AI makes stuff up sometimes. If AI is going to be used more and more then how are we going to manage this? I’m really worried—well, you could easily see AI replacing humans, right? I read about AI chatbots being used for smoking-cessation counselling instead of pharmacists, but who is monitoring what it is saying or doing? We need some kind of rules so everyone knows what they’re supposed to do.”*

*“At first it’s like—wow! It’s amazing, so cool, so powerful what it can do. But then when you use AI for a while, you start to sense some of the problems, the issues. What about climate change, right? AI uses ridiculous amounts of electricity. What about documentation—no one has any idea what we’re supposed to be charting. Someone needs to give us the rule book here so the pharmacists can actually use AI properly, safely.”*

All participants in this research indicated there was an urgent need for more education and greater regulatory vigilance around adoption of AI in pharmacy practice. Most participants described themselves as “naïve” or “inexperienced” with respect to AI and related technologies, but understood there were potential risks and harms from its use of which pharmacists needed to be made aware. Most participants felt that it should be the primary responsibility of licensing and regulatory bodies to authorize the use of specified AI products by pharmacists, rather than the current free-for-all situation where individuals needed to make this decision for themselves. The notion of a central authority with the expertise to fully evaluate safety and efficacy of AI products, then generate a list of “approved” AI products that could be effectively adopted in regulatory practice was universally endorsed by participants in this study, and identified as an essential patient-safety safeguard, one that was also important to protect pharmacists from inadvertently or unintentionally misusing the technology.

Specific areas for regulation and guidance identified by all participants in this study included the following:

(A) Privacy and Confidentiality: All participants in this study were familiar with existing legislation and regulation regarding safekeeping of confidential patient information and sensitive health data. All participants were also aware (to some degree) of the complexities associated with modern technologies, especially cloud-based computing applications including AI. Concerns were expressed about the disposition of confidential data when AI was being used, and what responsibilities pharmacists might have in this regard. Interestingly, no participants in this study connected this concern to their own use of personal devices (such as mobile phones with GoogleLens or ChatGPT) being used to answer professional questions. Participants in this study indicated that regulatory control or guidance to reassure both patients and pharmacists of safe transmission, storage, and disposition of personal health information was necessary.
(B) Consent and Disclosure Requirements: Most participants in this study expressed confusion regarding their responsibilities to disclose the use of AI in clinical decision making or other practice activities, and whether or not explicit informed consent was required or expected. All participants recognized the value of consent and disclosure provisions, but were uncertain as to whether this was applicable to the use of AI—and if so, in what situations, and contexts disclosure and consent should be required. Confusion further extended to the question of whether it was an expectation that patients be given a choice as to whether or not AI was used in their care or interaction with the pharmacist, an option that did not appear feasible to most participants.
(C) Managing Disagreements: The problem of hallucinations with AI was well-understood by all participants in this study, though the mechanisms or reasons behind hallucinations were not well described. For most participants in this study, the concern regarding hallucinations highlighted regulatory questions with respect to managing situations where AI may make a recommendation with which the pharmacist may disagree. Managing such disagreement extended to questions regarding whether disclosure of the disagreement to a patient was desired or required, whether and how such disagreement ought to be documented, and what process and workflow implications may exist in such a situation.
(D) Managing Errors/Liability: Further aligned with the concerns associated with hallucinations, participants in this study expressed concerns regarding how to manage errors where AI made erroneous or potentially harmful recommendations and whether the pharmacist bore some or all of the liability for following AI guidance. While all participants in this study accepted and acknowledged their own professional responsibilities and ethical obligations, they also raised questions regarding liability for errors, how this might be managed through a complaints-and-disciplinary process at a regulatory body, and what civil liability issues may exist where erroneous AI recommendations were accepted and followed.
(E) Documentation Expectations: Recently, there has been considerable emphasis on enhancing the quality and depth of clinical documentation by pharmacists. Participants in this study recognized there is potential value to AI-supported documentation (for example, through use of AI-driven ambient listening tools), but also noted there was limited or no regulatory guidance with respect to expectations. Questions arose with respect to the degree to which oversight and review of AI-generated documentation was required, and whether such oversight negated the time-saving benefit of using AI in the first place.
(F) Workflow Adaptations: All participants in this study understood the potential transformational power of AI in pharmacy practice and expressed support for technologies that could enhance patient safety, improve operational efficiency, and reduce costs. All participants expressed concerns regarding how AI might be integrated into pharmacy practice—would it actually replace human workers (pharmacists or technicians) or complement them? If complementing the human workforce, how would it be incorporated into existing workflows in a way that was human-centred and productive? Many participants had previous experiences of other non-AI technologies in pharmacies (for example, robotic dispensing systems) and highlighted numerous operational issues that were poorly addressed (including how to handle power failures or technology breakdowns, or changes in vendors and lack of ability to find suitable service agents to provide support). These experiences heightened participants’ concerns about growing reliance on AI, particularly in the context of the potential downgrading of professional expertise that could result.

Other issues that were raised by a small number of participants included climate-change-related impacts of AI use and the profession’s responsibility to mitigate climate-related harm, concerns regarding patients completely circumventing health professionals and simply relying entirely on AI for health-related advice, and concerns regarding mass de-skilling of the pharmacy workforce as it grows ever more reliant on AI.

A dominant concern expressed by all participants in this study was the lack of clarity, guidance, education or support in the pharmacy community to help guide responsible adoption of AI in practice. Participants expressed confusion regarding who should be asked such questions, whose responsibility it was to address these issues, and how they could be expected to proceed to responsibly adopt AI as professionals, with minimal support from leaders in the profession. One participant described this as the “Wild West”, a situation where everyone was “doing their own thing and hoping for the best”—a situation that appeared both unprofessional and ripe for problems.

*Theme 3: In the absence of regulation, ad hoc informal decision making is occurring.*

*“So that’s where we are at. Unless someone—and I think it really has to be the regulatory bodies that license pharmacists and accredit pharmacies—unless they step up and give us the rules to follow, it’ll be chaos and this might be a real risk to patients.”*

*“We are all just improvising right now because no one in leadership is actually giving us a rule book for how to use AI. Okay, without that rule book then, I guess we just have to expect bad stuff will happen? I really don’t like that and think it’s so unnecessary and just wish someone would help us figure out how to use this amazing technology in a safe way.”*

*“Isn’t this the job of the regulatory bodies? They make sure we are competent to be pharmacists, and it’s their job to inspect pharmacies to make sure everything is safe for patients. They are the ones who are in the best position I think to just make rules so everyone knows how to use AI. Even if this means—I don’t know—like make us take a special course or get a certificate or something to prove we are using AI correctly?”*

A strong theme from this research was participants’ belief that regulators (licensing bodies and accreditation groups) had primary responsibility for developing rules, guidance, and education to support responsible adoption of AI by pharmacists in practice. The need for greater involvement of centralized authorities like regulators was deemed by all participants to be both the most practical, and perhaps the only, path forward to ensure safe and effective use of AI by pharmacists in the public interest. In the current environment of minimal involvement by regulators and accreditors, concerns were expressed that this was leading to concerning ad hoc decision making that may prioritize certain factors (e.g., cost-saving, managing staffing shortages) at the expense of safe and effective patient care and confidentiality of patients’ health data.

All participants in this study framed this as a binary choice: regulate clearly or expect unfortunate consequences and outcomes. Participants rejected the assertion that pharmacists—as trained and licensed professionals—ought to educate themselves and align their decisions regarding AI in pharmacy with existing standards of practice and codes of ethics. Participants expressed their belief that pharmacists may be medication-therapy experts but that they are neither computer scientists nor data engineers, and therefore could and should not be expected to have the sophistication to analyze existing commercially available products and answer questions such as those posed in Theme 2 above. Instead, they expressed their belief that this is a regulatory and accreditation responsibility—and once rules were developed, then it would become the responsibility of the pharmacist to comply.

Participants framed this concern in the context of well-publicized negative experiences of recent years with large technology companies and social media platforms (such as “X” and Facebook) which have generated distrust and misapprehension regarding corporate responsibility for technology products. While most participants expressed their desire to ensure technological development and innovation of pharmacy-specific AI could proceed, they equally prioritized safety and efficacy for patients and pharmacists. Participants noted a potential role for regulators to proactively engage with technology companies and entrepreneurs, rather than reactively regulate once products had been developed and sold. Recognizing the challenge associated with reactive regulation in the context of enormous multinational technology companies, the value of engaging them early and ensuring pharmacists’ and patients’ perspectives and interests were introduced at the design phase was endorsed by most participants as a particularly valuable option and opportunity.

Several participants commented at length about potential risks of continued regulatory ambiguity with respect to AI in pharmacy practice, connecting recent experiences with Facebook and X to potential risks to patients. Few participants felt educational institutions, professional advocacy groups, unions, employers, hospitals, health systems, or other parties in the pharmacy profession had the power or scope to influence development of AI to the same degree as regulators. They also felt that regulators could convene “coalitions of the willing” and help mobilize different parts of the profession to work collaboratively to define guardrails for safe and responsible AI that could then be used by entrepreneurs and technology companies to actually develop products.

Failure of regulators to engage and act—particularly at this point in time, when AI is rapidly evolving but still relatively new—was described by most participants as a significant risk for both patients and the profession. For several participants, it was described in somewhat existential terms, particularly with respect to the potential of AI to replace the human workforce, and the risk of AI eclipsing human capacity to control or contain it. While other participants acknowledged potential risks, this was less catastrophic, and more focused on ensuring AI could achieve its fullest potential in complementing the human workforce, rather than replacing or displacing it.

## 4. Discussion

Findings from this study highlight important issues for regulators, employers, educators, professional associations, and the entire pharmacy profession. The picture painted by this data is one of unstandardized and somewhat random use of a power technology by pharmacists, who may or may not be aware of implications ranging from privacy/confidentiality of data, to the quality of the information itself. In the absence of formal regulation or rules governing the use of AI in pharmacy practice, pharmacists are using their best judgement—based on their own inexpert understanding—to leverage advantages of this rapidly evolving technology. The objective of this study was not focused on identifying or characterizing potential risks or harms associated with this unstandardized use of AI by pharmacists, though this may be a valuable study to consider in the future. Based on the interviews undertaken, there is no data to suggest actual harm or risk to patients has occurred or has been experienced. Still, the pharmacists involved in this study themselves described personal discomfort and professional dissatisfaction at the absence of a regulatory framework to guide responsible adoption of AI in practice. All participants in this study recognized the potential value and power of AI in transforming and augmenting the work of pharmacists and the role of pharmacy, and no participants believed or suggested that innovation in AI ought to be discouraged or reduced. Instead, they sought guidance from external, reliable sources—in particular, licensing and regulatory bodies—to safely guide responsible adoption of AI in practice. All participants expressed openness at using AI to support their work, particularly human-in-the-loop or human-on-the-loop AI.

Most participants in this study readily acknowledged their personal limitation in understanding and using the technology—for example, during interviews, many participants were unaware that apps available and used on their personal cellphone (such as GoogleLens) were themselves powered by AI technologies. Few participants discussed issues related to climate change that may be accelerating due to the computational power required to run AI-based systems. Few participants described potential privacy or other risks associated with using personal devices in the context of professional work. This suggests there are significant opportunities—and needs—for focused education for pharmacists to support their fuller understanding of the technology itself, and its potential implications.

The consensus amongst participants was that primary responsibility for supporting pharmacists in this area and for ensuring responsible adoption of AI in practice should rest with the licensing and regulatory bodies responsible for certifying pharmacists and accrediting pharmacies. Most participants rationalized this in the context of the regulator’s responsibility to protect patients and ensure safe and effective practice of the profession. No participants acknowledged potential risks and complexities for regulators in assuming this role—importantly, none of the participants in this study had previous experience working in a regulatory body, so may not be familiar with the issues faced by regulators in assuming new responsibilities such as this. Instead, participants in this study favoured legalistic approaches to regulatory practice to support pharmacists: for example, accrediting pharmacy-specific AI for use in practice, or mandating education programs for pharmacists using AI. This legalistic approach contrasts with the regulators’ own sentiments described in previous research [26,27]. In that research, regulators expressed strong support for principles-based “guidance” rather than rules and regulations, indicating that a rules-based approach to AI would likely be unsuccessful. Reasons given by regulators for concerns regarding viability of a rules-based approach included their own acknowledgement that they lacked the skills and competencies to truly understand the technology, that AI itself was evolving so rapidly and as a result would always outpace the ability of regulators to issue rules to govern it, and concerns that such heavy-handed approaches might stifle innovation.

Taken together, these findings suggest a misalignment between the expressed needs and wishes of pharmacists for greater regulatory control and involvement in AI, with a reluctance of regulators to use their strongest, legalistic powers in this context. This misalignment raises important questions regarding current and future use of AI by pharmacists, and suggests that, in the near future, there may be on-going unstandardized and somewhat random use of AI by pharmacists until some group (employers, professional associations, or educators) steps into the void to assume greater leadership in supporting or directing the profession’s use of this technology. This highlights an important issue for pharmacy leaders across all sectors to consider: AI represents a potentially significant pivot for the profession itself, yet the lack of coordination with the profession itself to mitigate risks and ensure responsible and appropriate adoption of the technology appears lacking. In this vacuum, AI will continue to evolve and will continue to be used by pharmacists—but in a way that may not be optimal for either patient care/safety or the profession’s own best interests.

Clearly, further work in this area is required, and consideration for how a profession like pharmacy can navigate forward must be prioritized. There is no evidence to suggest other health professions (such as medicine or nursing) have developed better or more sophisticated approaches to the adoption of AI; it appears likely that similar patterns of non-standardized adoption by clinicians may be occurring in those professions. Determining how best to adopt AI in clinical practice may require an interprofessional, trans-jurisdictional approach to allow resources of multiple professions to be focused on the issue. In the absence of discussions or plans on how best to proceed, the technology continues to evolve and be used/adopted, but in ways that may be uncomfortable, sub-optimal, and not necessarily aligned in the best possible way with patients’ or professionals’ best interests.

There are important limitations to consider for this study. The exploratory methodology used was appropriate, given the scant available evidence published in the literature concerning the central question of how pharmacists are using AI in practice. However appropriate, this methodology required a focus on a small group of pharmacists in a single jurisdiction, and, therefore, generalizability beyond this cohort cannot be assumed nor inferred. The recruitment method for participants may have led to an unrepresentative sample of participants; those with interests or some sophistication in using AI were the ones most likely to be interested enough to respond and enroll in the study, so these participants are likely not representative of the population of pharmacists in Ontario. As a result, caution is required in generalizing findings of this study to the population of pharmacists in Ontario, Canada, or elsewhere, as the study cohort likely had higher awareness and motivation to engage with this study than average pharmacists. The narrative style of semi-structured interviews presumes a certain degree of authenticity, self-awareness, and accuracy in reporting; it was not possible to confirm statements or observe specific behaviours described by participants in this study, so their self-reporting was taken at face value. Attempts to mitigate some of these potential limitations included use of the COREQ checklist in an attempt to enhance trustworthiness—specific techniques such as member-checking, interviewing until thematic saturation, and independent coders using constant-comparative analytic techniques are accepted methods for enhancing quality in qualitative research. Finally, the speed of evolution of AI itself means that, in some ways, findings from a study such as this may already be outdated, as the pace of change in the technology reshapes individual behaviours, both personally and professionally. Still, this study presents an interesting perspective for stakeholders across the profession, and points to a potentially important misalignment between pharmacists’ expectations and the approach being taken by the profession in managing one of the most significant issues facing pharmacy—and society—today. Further work examining regulators’ perspectives on this misalignment, as well as the perspectives of employers, technology entrepreneurs, and patients, will be important to determine pathways to create greater alignment of understanding and action within and across the pharmacy profession.

## 5. Conclusions

Artificial intelligence will continue to reshape our world—and the practice of pharmacy—in the years ahead. Despite the magnitude of change that may be upon us, there currently appears to be very limited coordination within and across the pharmacy profession with respect to supporting pharmacists in responsibly adopting these technologies. As highlighted in this study, this is resulting in pharmacists experimenting and using AI in an unstandardized and somewhat random manner. For participants in this study, this is a matter of concern that leads them to believe someone—most likely regulators/licensing bodies/accreditors—ought to be leading a coordinated and evidence-based response to support the pharmacy profession. Whether this is possible or not was not evaluated in this study. Further work is required to determine how best to safeguard patients’ interests as well as the interests of pharmacists and the profession itself. The transformational potential of AI—both in professional and everyday life—is staggering. What will actually happen as the technology continues to evolve, spread, and shift towards human-out-of-the-loop variants will be a significant challenge for all of us.

## Figures and Tables

**Table 1:** Demographic Characteristics of Research Participants (*n* = 24).

Participant Characteristics (*n* = 24)		
Sex	Male = 10	Female = 14
Age	Mean = 43.8 years old (range = 25 to 61 years old)	
Years working in community pharmacy practice	Mean = 18.1 years (range = 2 to 36 years)	
Geographic location of pharmacy practice	Large urban: 8 Mid-sized city: 4 Suburban: 6 Small town: 3 Aboriginal/indigenous: 2	
Type of pharmacy practice	Independent owner-operator: 6 Chain pharmacy: 15 Big-box retailer/Grocery: 3	
Previous experience working in licensing, regulatory or accreditation body	No = 24	Yes = 0
Previous experience using AI-powered technology in pharmacy practice	Yes = 24	No = 0
Formal certification, education or training in use of AI-powered technology	No = 24	Yes = 0

**Table 2:** Semi-structured interview protocol (final version, following pilot-testing and modifications).

A. Introduce self and affiliation.
B. Confirm identity of participation by name and affiliation.
C. Request permission to record interview using Zoom platform. If “yes”, record. If “no,” take field notes as required.
D. Review study background information. Confirm participant’s understanding of study and confidentiality provisions. Provide opportunities for questions and clarifications. Receive positive, explicit verbal consent to proceed with interview and study.
Prompt 1: Can you tell me about your role at [insert name of participant’s pharmacy or organization]? Can you tell me about the communities you serve?
Prompt 2: What is your understanding of artificial intelligence and how it functions? How important is this issue to you, personally and professionally?
Prompt 3: How has your pharmacy team been discussing the issue of artificial intelligence in pharmacy practice?
Prompt 4: How have you been using artificial intelligence in your pharmacy? How have you been learning to use it?
Prompt 5: How have other pharmacists in your practice or pharmacists you may know been using artificial intelligence in their day-to-day work? How have they been learning to use it?
Prompt 6: What actions or steps are being taken in your practice to ensure responsible, safe, and effective adoption of AI by pharmacists? How successful or effective have these actions or steps been? What more could or should be done, from your perspective?
Prompt 7: What actions or steps do you see being taken by the profession of pharmacy to ensure responsible, safe, and effective adoption of artificial intelligence by pharmacists? How successful have these been? What has worked and what has not worked? [sub-prompt: Why do you think this has worked or not worked?]
Prompt 8: What do you feel are the most impactful activities or steps pharmacists and the profession can take with respect to artificial intelligence? What are the key barriers and facilitators to these activities and steps?
Prompt 9: Is there anything else we have not discussed today that you would like to bring up?
E. Thank participant for opportunity to speak with them.
F. Remind participant they are free to review transcripts once available, in one week.
G. Ask participant if they have any questions, concerns or feedback about the interview.
H. Indicate recording of interview will now stop. Stop recording.
I. Thank participant for involvement, and conclude interview.

## Data Availability

The original contributions presented in this study are included in the article. Further inquiries can be directed to the corresponding author.

## Abbreviations

The following abbreviations are used in this manuscript:

AI	Artificial Intelligence
HiL	Human-in-the-loop
HoL	Human-out-of-the-loop
HonL	Human-on-the-loop
GIGO	Garbage in, Garbage Out
COREQ	Consolidated criteria for reporting qualitative research

## References

[1] Bajwah J., Munir U., Nori A., Williams B. (2021). Artificial intelligence in healthcare: Transforming the practice of medicine. Future Healthc. J..

[2] Angus D.C., Khera R., Lieu T., Liu V., Ahmad F.S., Anderson B., Bhavani S.V., Bindman A., Brennan T., Celi L.A. (2025). AI, health, and Health Care Today and Tomorrow: The JAMA Summit Report on Artificial Intelligence. JAMA.

[3] Hirani R., Noruzi K., Khuram H., Hussaini A.S., Aifuwa E.I., Ely K.E., Lewis J.M., Gabr A.E., Smiley A., Tiwari R.K. (2024). Artificial intelligence and healthcare: A journey through history, present innovations, and future possibilities. Life.

[4] Jiang F., Jiang Y., Zhi H., Dong Y., Li H., Ma S., Wang Y., Dong Q., Shen H., Wang Y. (2017). Artificial intelligence in healthcare: Past, present, and future. Stroke Vasc. Neurol..

[5] Miotto R., Wang F., Wang S., Jiang X., Dudley J.T. (2018). Deep learning for healthcare: Review, opportunities and challenges. Brief. Bioinform..

[6] Sendekie A.K., Limenh L.W., Abate B.B., Chanie G.S., Kassaw A.T., Tamene F.B., Gete K.Y., Dagnew E.M. (2024). Artificial intelligence in community pharmacy practice: Pharmacists’ perceptions, willingness to utilize and barriers to implementation. Explor. Res. Clin. Soc. Pharm..

[7] Tai M. (2020). The impact of artificial intelligence on human society and bioethics. Tzu Chi Med. J..

[8] Spiegler S., Hoda R., Pant A. (2026). Images of AI: How AI practitioners view the impact of artificial intelligence on society, now and in the future. Technol. Soc..

[9] Wells B.J., Nguyen H.M., McWilliams A., Pallini M., Bovi A., Kuzma A., Kramer J., Chou S.-H., Hetherington T., Corn P. (2025). A practical framework for appropriate implementation and review of artificial intelligence (FAIR-AI) in healthcare. npj Digit. Med..

[10] Sargsyan A., Hovsepyan S., Muradyan A., Kozlakidis Z., Muradyan A., Sargsyan K. (2024). Ubiquitous and Powerful Artificial Intelligence. Digitalization of Medicine in Low- and Middle-Income Countries.

[11] Raza M., Aziz S., Noreen M., Saeed A., Anjum I., Ahmed M., Raza S.M. (2022). Artificial intelligence in pharmacy: An overview of innovations. Innov. Pharm..

[12] Shishehgar S., Murray-Parahi P., Alsharaydeh E., Mills S., Liu X. (2025). Artificial intelligence in health education and practice: A systematic review of health students’ and academics’ knowledge, perceptions, and experiences. Int. Nurs. Rev..

[13] Weber S., Wyszynski M., Godefroid M., Plattfaut R., Niehaves B. (2024). How do medical professionals make sense (or not) of AI? A social-media-based computational grounded theory study and an online survey. Comput. Struct. Biotech. J..

[14] Giebel G., Raszke P., Nowak H., Palmowski L., Adamzik M., Heinz P., Tokic M., Timmesfeld N., Brunkhorst F., Wasem J. (2025). Problems and barriers related to AI-based clinical decision support systems: Interview study. J. Med. Internet Res..

[15] Panteli D., Adib K., Buttigieg S., Goiana-Da-Silva F., Ladewig K., Azzopardi-Muscat N., Figueras J., Novillo-Ortiz D., McKee M. (2025). Artificial intelligence in public health: Promises, challenges, and an agenda for policy makers and public health institutions. Lancet Public Health.

[16] Tawfik D., Sinha A., Bayati M., Adair K.C., Shanafelt T.D., Sexton J.B., Profit J. (2021). Frustration with technology and its relation to emotional exhaustion among health care workers: Cross sectional observational study. J. Med. Internet Res..

[17] Hatem R., Simmons B., Thornton J.E. (2023). A call to address AI hallucinations and how healthcare professionals can mitigate their risks. Cureus.

[18] Strack R. (2026). The AI sycophant. Nat. Biomed. Eng..

[19] Federspiel F., Mitchell R., Asokan A., Umana C., McCoy D. (2023). Threats by artificial intelligence to human health and human existence. BMJ Glob. Health.

[20] Rendle K.A., Abramson C.M., Garrett S.B., Halley M.C., Dohan D. (2019). Beyond exploratory: A tailored framework for assessing qualitative health research. BMJ Open.

[21] De Jonckheere M., Vaughn L.M. (2019). Semi-structured interviewing in primary care research: A balance of relationship and rigour. Fam. Med. Community Health.

[22] (2022). Equator Network (Enhancing the Quality and Transparency of Health Research) Reporting Guidelines. https://www.equator-network.org/reporting-guidelines/coreq/.

[23] Hewitt-Taylor J. (2001). Use of constant comparative analysis in qualitative research. Nurs. Stand..

[24] Saunders B., Sim J., Kingstone T., Baker S., Waterfield J., Bartlam B., Burroughs H., Jinks C. (2017). Saturation in qualitative research: Exploring its conceptualization and operationalization. Qual. Quant..

[25] Grootendorst P., Kralj B., Sweetman A. (2025). Long-term trends in the labour supply and productivity of pharmacists in Canada. Can. Pharm. J..

[26] Van der Gaag A., Jago R., Gallagher A., Stathis K., Webster M., Austin Z. (2023). Artificial intelligence in health professions regulation: An exploratory qualitative study of nurse regulators in three jurisdictions. J. Nurs. Regul..

[27] Bechtel M. (2024). Artificial intelligence: An emerging challenge for state medical boards. J. Med. Regul..

